# Development of a radiomics-vision transformer fusion model based on chest CT for predicting adverse respiratory events during recovery in elderly hip fracture patients under general anesthesia

**DOI:** 10.3389/fmed.2026.1907086

**Published:** 2026-08-05

**Authors:** Jiasen Hu, Yuxuan Wu, Jiancai Lin, Xuewen Chen

**Affiliations:** 1 Department of Orthopaedics, Yueqing People’s Hospital, Wenzhou, Zhejiang, China; 2 School of Mental Health, Wenzhou Medical University, Wenzhou, Zhejiang, China; 3 Department of Orthopaedics, The Second Affiliated Hospital and Yuying Children’s Hospital of Wenzhou Medical University, Wenzhou, Zhejiang, China

**Keywords:** adverse respiratory events, deep learning, elderly patients, general anesthesia, hip fracture

## Abstract

**Background:**

Hip fracture is a common and serious injury in the elderly. With the aging of the global population, the incidence of hip fracture is increasing. Adverse Respiratory Events (AREs) are common in elderly patients with hip fracture during recovery from general anesthesia, which can lead to serious complications. However, current methods for predicting these events are limited.

**Methods:**

This retrospective multicohort study analyzed clinical data from 664 patients across two institutions. Radiomic features were extracted from regions of interest (ROIs) in chest CT scans, and deep learning features were extracted using a vision transformer (ViT) model. A radiomics-ViT fusion model was developed by combining these features. The performance of the models was evaluated using metrics such as area under the curve (AUC), sensitivity, specificity, and F1-score.

**Results:**

The radiomics-ViT fusion model demonstrated excellent performance, with an AUC of 0.994 in the internal training set and 0.875 in the external test set. This was significantly better than the XGBoost model (AUC 0.553) and the ViT model alone (AUC 0.788) in the external test set. The fusion model accurately identified high-risk patients, enabling timely interventions and improved outcomes.

**Conclusion:**

The developed radiomics-ViT fusion model serves as a valuable tool for predicting AREs during recovery in elderly hip fracture patients under general anesthesia, enhancing clinical decision-making and patient care.

## Introduction

Hip fractures represent a common and serious injury among the elderly, with their incidence rising as the global population ages (1). Nearly all patients with hip fractures undergo surgical treatment, which requires regional neuraxial block or general anesthesia using systemic anesthetic agents (2). The management of the recovery period after general anesthesia is particularly critical in elderly patients with hip fracture (3). Adverse Respiratory Events (AREs) are common in this stage, including upper airway obstruction and hypoxemia (SpO_2_ < 90%), which may lead to severe complications, prolonged hospital stay, and increased medical burden (4). However, the current prediction methods for these adverse respiratory events are still limited, and there is a lack of accurate and objective assessment tools. Timely identification of high-risk individuals following hip fracture surgery enables early medical intervention, ultimately improving patient prognosis (5, 6).

Many studies have shown that the lung tissue status of patients during the perioperative period is closely related to postoperative recovery (7, 8). Patients with compromised lung tissue are at an increased risk of pulmonary complications like pneumonia, pulmonary edema, and respiratory failure. These complications can lead to prolonged hospitalization, heightened care requirements, and adverse effects on overall recovery (9, 10). Consequently, chest CT scans are routinely administered to assess lung conditions in elderly hip fracture patients before anesthesia and surgery.

With the rapid development of artificial intelligence (AI) technology, AI models are increasingly being employed in the medical field (11–14). Radiomics, a technique that extracts and analyzes numerous features from medical images, aims to uncover disease - related information. Radiomics features can be extracted from lumbar muscle CT images to construct a machine learning model for effective osteoporosis patient screening (15). The Vision Transformer (ViT) model, an advanced machine learning technique, excels in processing 3D medical images, such as in community - acquired pneumonia diagnosis (16).

The aim of this study was to develop a radiomics-ViT fusion model based on lung tissue images from chest CT for predicting the risk of adverse respiratory events during recovery from hip fracture in elderly patients under general anesthesia. By integrating the unique advantages of radiomics and ViT models, this study is expected to provide clinicians with a more reliable, data-driven tool to optimize post-anesthetic management and enhance the identification of high-risk patients, thereby improving patient outcomes and optimizing medical resource allocation.

## Materials and methods

### Study design

This retrospective multicohort study analyzed clinical data from patients at the Second Affiliated Hospital of Wenzhou Medical University (Institution 1) and Yueqing People’s Hospital (Institution 2) between 1 January 2024 and 31 December 2024. Institutional review boards (IRBs) at both sites reviewed and approved the study protocol. Given its observational nature and use of de-identified data, the IRBs waived the requirement for written informed consent.

For this study, a retrospective collection of 664 patients from two institutions was conducted. All the patients were extubated in the operating theater and transferred to PACU (Postanesthesia care unit) without oxygen. The inclusion criteria were as follows: (1) age ≥ 60 years and (2) hip fracture diagnosis confirmed by X-ray or CT examination. The exclusion criteria were: (1) no chest CT examination performed, (2) CT image artifacts, (3) refusal of surgery, (4) non-general anesthesia, and (5) requirement of other surgeries. Institution 1 enrolled 436 patients, and Institution 2 included 228 patients. The study population was divided into an AREs group and a non-AREs group according to the occurrence of AREs during the recovery period after anesthesia. AREs were defined as any of the following during the postoperative hospital stay: (1) including upper airway obstruction that requires an intervention, (2) SpO_2_ less than 90% on nasal oxygen (3 L/min) and intubation.

**FIGURE 1:**
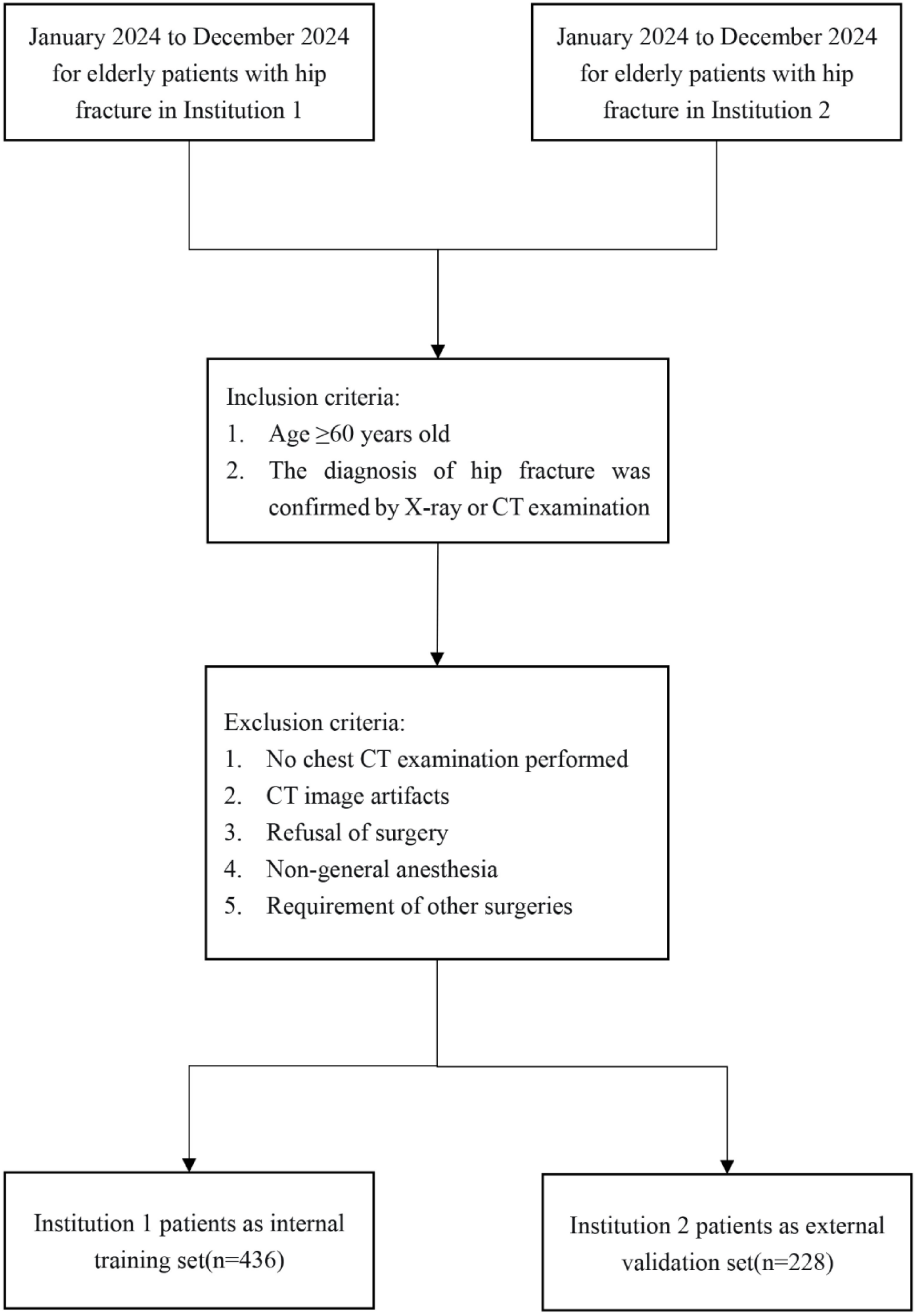
The recruitment process of patients in this study. CT, computed tomography; ICU, intensive care unit.

**FIGURE 2:**
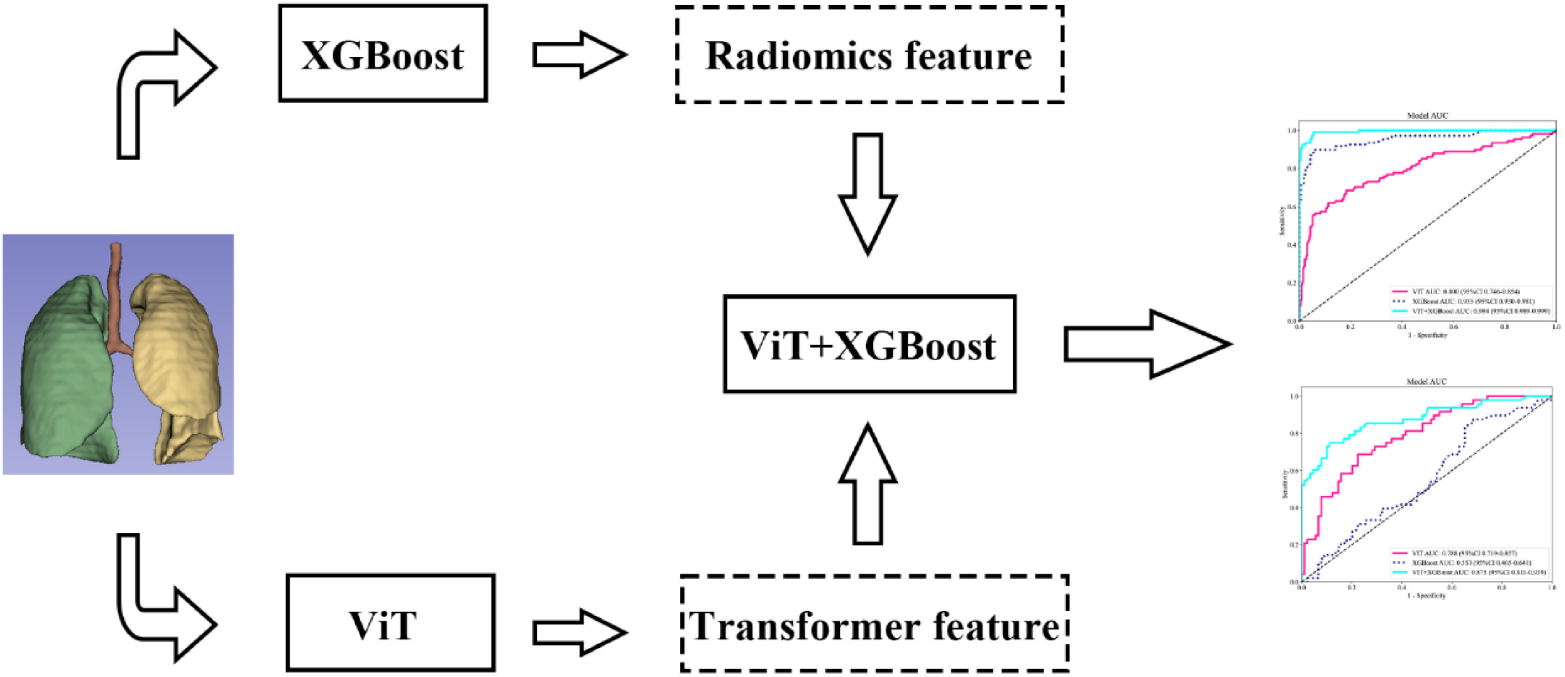
The research process of this study. ViT, vision transformer.

The study flowchart (Figure 1) details patient selection, while Figure 2 outlines the methodological framework.

### Data preprocessing and annotation

Unenhanced chest CT scans (5 mm slice thickness) were retrieved from PACS systems. Images were resampled to 1 × 1 × 1 mm^3^ voxel spacing and standardized to window level 40 and width 300 for consistent visualization.

Regions of interest (ROIs) comprising tracheal and lung tissues were delineated by an expert clinician using the Chest Imaging Platform (3D Slicer v5.2.2) and validated by a senior clinician. All annotators were blinded to patient identifiers.

### XGBoost radiomics model

Unenhanced chest CT ROIs were imported into PyRadiomics to extract standardized radiomic features following the Image Biomarker Standardization Initiative (IBSI) protocol. Seven feature families were generated: first-order statistics, morphological shape features, gray-level co-occurrence matrix (GLCM), gray-level run-length matrix (GLRLM), gray-level size-zone matrix (GLSZM), gray-level dependence matrix (GLDM), and wavelet-filtered texture features. All raw radiomic features were standardized via z-score normalization to eliminate magnitude bias before downstream filtering. A three-step feature selection pipeline was strictly executed only on the internal training cohort to avoid data leakage: (1) Univariate screening: Mann–Whitney U test retained features with inter-group difference *p* < 0.05. (2) Collinearity elimination: Spearman correlation threshold set at 0.9; for each highly correlated feature pair, only the feature with smaller *p*-value was preserved. (3) Sparse feature screening: LASSO logistic regression with 10-fold internal cross-validation. The optimal regularization coefficient λ was determined by the minimum mean cross-validated loss to obtain the final predictive radiomic signature. All selected radiomic features were fed into an XGBoost classifier to predict postoperative AREs. Feature filtering and model training were conducted exclusively on the internal training cohort (Institution 1), while the external test cohort (Institution 2) remained fully unseen throughout model development and was only used for final generalization performance evaluation.

### 3 D vision transformer (ViT) model

The ViT model processes input ROIs sized 64 × 64 × 48 voxels. Images are partitioned into 16 × 16 × 2 patches, flattened, and linearly embedded into a 1,024-dimensional vector space with positional embeddings to preserve spatial context.

The architecture comprises six transformer encoder layers, each with multi-head self-attention (eight heads) and a feed-forward network (2,048 dimensions). Dropout (rate 0.1) was applied to prevent overfitting.

Model training configuration: Adam optimizer with initial learning rate = 0.001, batch size = 4, maximum training epoch = 600. Cosine annealing learning rate decay was applied for dynamic learning rate adjustment. Multi-modal volumetric data augmentation (random rotation, scaling, elastic deformation, intensity shift) was implemented during training to improve generalization. An early stopping strategy (patience = 50 epochs, monitored by validation loss) halted training if no performance improvement was observed to prevent overfitting.

After training convergence, high-dimensional deep learning embeddings were extracted from the final transformer encoder layer and normalized via z-score transformation. Consistent with the radiomics pipeline, these deep features were fed into an XGBoost classifier for standalone ViT prediction. The classifier was trained on the internal training cohort (Institution 1), while Institution 2 served as a fully independent external test cohort with no involvement in any step of model training, feature selection or hyperparameter optimization. This unified classifier framework across all models ensures a controlled, fair comparative analysis.

### ViT+XGBoost fusion model

Normalized radiomic signature features and ViT deep embeddings were concatenated into a unified high-dimensional fused feature set. The same two-step feature pruning workflow was applied exclusively to the internal training cohort (Institution 1): Spearman correlation filtering (r > 0.9) followed by LASSO regression with 10-fold cross-validation to screen the optimal fused predictive feature subset.

The final fusion prediction model was built using the identical XGBoost framework and hyperparameter tuning strategy described above, with all model training and parameter optimization performed solely on Institution 1. Institution 2 served as a fully independent external test cohort and was not involved in any step of feature selection, hyperparameter tuning or model fitting. This consistent modeling framework eliminates classifier heterogeneity as a confounding variable when comparing model performance, and the fully held-out external validation design ensures reliable assessment of cross-center generalizability.

### Statistical analysis

The Shapiro-Wilk test was applied to assess the distribution of clinical baseline characteristics. The Mann-Whitney U test was used for continuous variables that were not normally distributed, and the Pearson Chi-square test was used for categorical variables. Continuous variables are presented as the mean ± standard deviation, and categorical variables are presented as percentages. Performance metrics, including sensitivity, specificity, F1-score, Delong test and the AUC (area under the curve), were used to evaluate the different models. Data were processed using SPSS (version 26.0; SPSS Inc., Chicago, IL, USA) and Python (version 3.9.7).

## Results

### Clinical baseline characteristics

A total of 664 elderly patients with hip fractures were included in this study, of which 436 were included in Institution 1 (internal training set) and 228 were included in Institution 2 (external test set). There were no significant differences between the two institutions in age, BMI, sex, or AREs. In addition, postoperative AREs occurred in approximately 24.8% of the patients at Institution 1 and in 21.5% of the patients at Institution 2. Detailed results are summarized in Table 1

**TABLE 1:** Clinical baseline characteristics of the participating populations at both institutions.

Variable	Internal training set (Institution 1, *n* = 436)	External test set (Institution 1, *n* = 228)	*P*-value
Age, (years)	79 ± 9	78 ± 10	0.517
BMI, (kg/m^2^)	21.93 ± 3.65	22.45 ± 3.44	0.078
Gender, *n* (%)			0.217
Female	306 (70.2)	150 (65.8)	–
Male	130 (29.8)	78 (34.2)	–
AREs, *n* (%)	108 (24.8)	49 (21.5)	0.310

BMI, body mass index; AREs, adverse respiratory events.

### Performance of the XGBoost model

**FIGURE 3:**
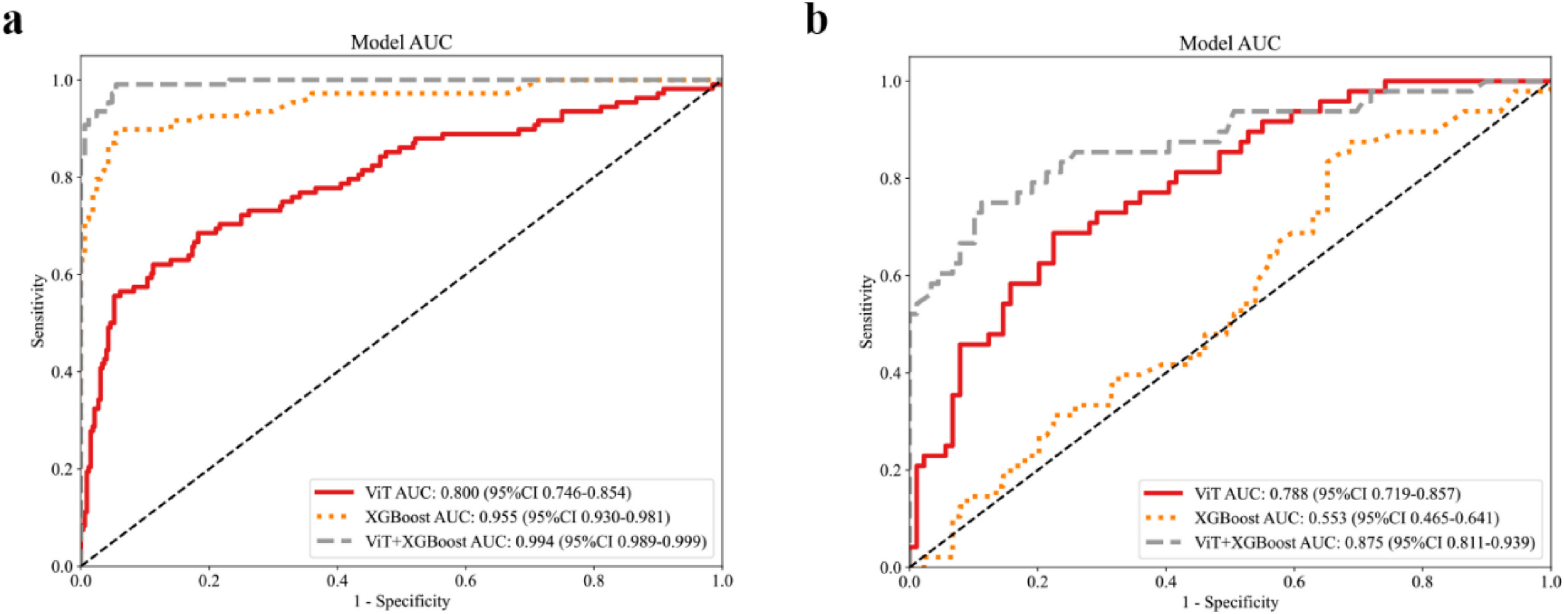
Receiver operating characteristic curves of different models in the **(a)** internal training set and **(b)** the external test set. ViT, vision transformer.

In this study, we evaluated the predictive utility of radiomics features for AREs in elderly patients with hip fractures. A total of 30 radiomics features were extracted and integrated into an XGBoost model. While the model demonstrated robust performance on the internal training set (AUC = 0.955, sensitivity = 0.870, specificity = 0.945, F1 score = 0.855), its generalizability was limited in external validation. Specifically, the XGBoost model achieved suboptimal accuracy in the external test set, yielding an AUC of 0.553 (sensitivity = 0.854, specificity = 0.315, F1 score = 0.389). These discrepancies highlight potential overfitting or heterogeneity between datasets. Detailed results are summarized in Table 2 and visualized in Figure 3.

**TABLE 2:** Predictive performance of different models.

Model	Dataset	AUC (95% CI)	Sensitivity	Specificity	F1 score
XGBoost	Train	0.955 (0.930–0.981)	0.870	0.945	0.855
	Test	0.553 (0.465–0.641)	0.854	0.315	0.389
ViT	Train	0.800 (0.746–0.853)	0.611	0.887	0.626
	Test	0.788 (0.719–0.857)	0.667	0.775	0.533
ViT+XGBoost	Train	0.994 (0.989–0.999)	0.972	0.945	0.909
	Test	0.875 (0.811–0.939)	0.729	0.888	0.680

AUC, area under curve; ViT, vision transformer.

### Performance of the ViT model

Compared with the XGBoost model, the ViT model has a certain improvement in the ability to predict AREs in elderly patients with hip fracture. In the internal training set, the AUC, sensitivity, specificity and F1 score of ViT model were 0.800, 0.611, 0.887, and 0.626, respectively. Similarly, in the external test set, the performance of the ViT model was somewhat degraded, with AUC, sensitivity, specificity, and F1 score of 0.788, 0.667, 0.775, and 0.533, respectively. Details are provided in Figure 3 and Table 2. In addition, 31 DL features were extracted from the final encoding layer in the ViT model.

### Performance of the ViT+XGBoost model

**FIGURE 4:**
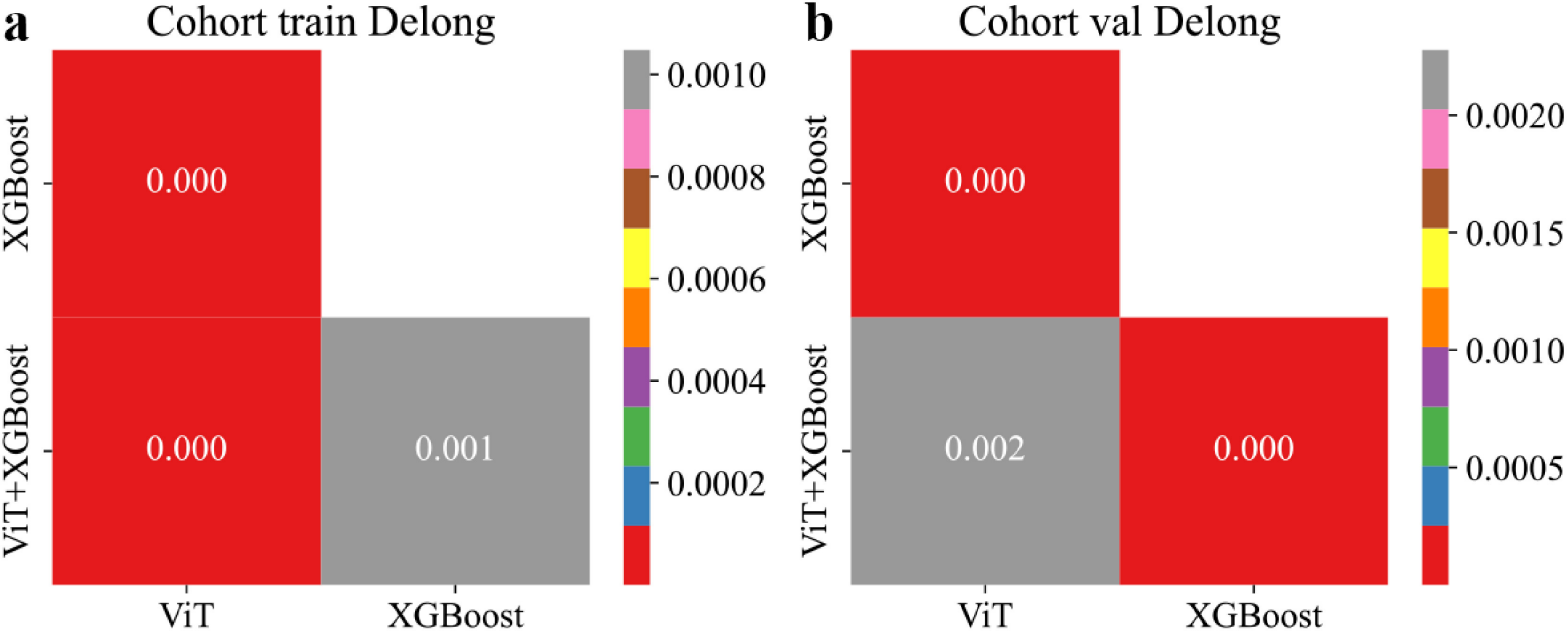
Delong’s test for different models in the **(a)** internal training set and the **(b)** external test set. ViT, vision transformer.

The fusion features (30 radiomics features and 31 deep learning features) screened by a series of statistical methods such as Mann-whitney U test and the Least absolute shrinkage and selection operator (LASSO) were used to construct ViT+XGBoost model to predict AREs in elderly patients with hip fracture. The prediction performance of the ViT+XGBoost model was excellent in both the internal training set and the external test set. The AUC, sensitivity, specificity and F1 score of the model were 0.994, 0.972, 0.945, and 0.909 in the internal training set, and 0.875, 0.729, 0.888, and 0.680 in the external testing set, respectively. The details are shown in Figure 3 and Table 2. In addition, Delong test showed that the AUC of ViT+XGBoost model was significantly higher than that of XGBoost model and ViT model in both training set and test set (Figure 4).

## Discussion

The aim of this study is to develop a radiomics-ViT fusion model based on chest CT lung tissue images for predicting the risk of AREs during recovery from hip fracture in elderly patients under general anesthesia. The results showed that the fusion model performed well on the internal training set and the external test set, with AUC values of 0.994 and 0.875, respectively, which were significantly better than the single XGBoost model (AUC of the external test set was 0.553) and ViT model (AUC of the external test set was 0.788). This indicates that the fusion model can more accurately identify high-risk patients, thereby providing clinicians with a more reliable decision support tool to optimize the management of anesthesia recovery period and improve patient prognosis.

Hip fracture is a common serious injury in the elderly, and the management of postoperative recovery period is very important to improve the prognosis of patients (17). AREs are common during recovery from general anesthesia. Timely detection of AREs will greatly improve the prognosis of patients. Accurate prediction of the risk of these events can help medical staff to take intervention measures in advance, optimize resource allocation, and reduce the incidence of adverse events.

The perioperative management of elderly hip fracture patients is significantly influenced by their lung condition. Preoperative pulmonary function serves as a crucial risk factor for postoperative complications in these patients (18). Anesthesiologists often rely on chest CT to assess lung tissue condition and determine if a patient can withstand surgery. Artificial intelligence models have the ability to detect image features that are hard for the human eye to discern, such as radiomics and enhanced deep learning features. These features may be closely linked to disease occurrence and prognosis.

Radiomics has been widely used in the medical field. For instance, an XGBoost model based on CT images can effectively predict lymph node metastasis in patients with head and neck cancer (19). Similarly, the Transformer model has numerous applications in 3D medical imaging, including the segmentation of pulmonary blood vessels and the screening of pancreatic masses (20, 21). In this study, XGBoost and Transformer models were constructed using lung tissue CT images to predict AREs in elderly patients with hip fracture during anesthesia recovery. However, both models showed poor performance in the external test set, with the XGBoost model achieving an AUC of 0.553 and the Transformer model an AUC of 0.788. Performance differences of models between internal and external cohorts are a common challenge in multicenter radiomics studies, driven primarily by several factors. First, feature selection itself is cohort-dependent. The feature screening process is developed on the training cohort, and the selected features may be better at capturing imaging characteristics specific to the internal population, leading to reduced predictive performance in independent cohorts with different baseline distributions. Second, subtle clinical phenotype differences. Even when baseline demographic characteristics are matched, variations in preoperative pulmonary comorbidities across centers, as well as differences in clinical intervention thresholds for AREs, may also contribute to diminished external performance. Surprisingly, when we developed a radiomics-Transformer fusion model, its performance in the external test set was significantly better than that of the XGBoost and Transformer models. This fusion model can more accurately identify high-risk patients, enabling timely medical intervention and enhancing patient prognosis. The improvement in the performance of the fusion model may stem from the complementarity of the features. The manually constructed radiological image features can capture the explicit textures and morphological patterns of the lung parenchyma, but they are highly sensitive to the heterogeneity of imaging protocols across different centers. In contrast, the ViT deep embedding can encode a more robust hierarchical spatial representation with greater robustness, and is more resistant to disturbances caused by changes in scanning parameters. By integrating the two complementary feature modalities, it can more comprehensively cover multiple pathological physiological dimensions related to perioperative respiratory dysfunction, rather than merely fitting the random noise in a specific cohort.

The radiomics-ViT fusion model developed in this study is able to provide clinicians with an objective and quantitative assessment tool to help them more accurately identify patients at high risk after surgery. By predicting the risk of adverse respiratory events in advance, medical staff can reasonably arrange postoperative nursing resources, optimize the treatment plan of patients, reduce unnecessary medical intervention, and improve the utilization efficiency of medical resources. In addition, the model can also provide new ideas and methods for clinical research and promote the further application of artificial intelligence technology in the medical field.

This study has several limitations. First, the retrospective study design carries an inherent risk of selection bias. Only elderly hip fracture patients who completed preoperative unenhanced chest CT were enrolled, which may exclude patients with milder baseline conditions who did not undergo routine CT examination, potentially leading to an overestimation of the incidence of AREs. Second, although two independent institutions were involved and provided preliminary cross-center evidence, the overall sample size is still relatively small, which limits the robustness of the selected fusion feature signature and requires further validation in a larger cohort. Third, although the model’s performance improved in the external test set, further verification on more diverse datasets is needed to confirm its stability and generalizability. Future large-scale prospective studies should be conducted to validate the model’s reliability.

## Conclusion

The radiomics-ViT fusion model developed in this study can effectively predict AREs during resuscitation in elderly patients with hip fracture under general anesthesia, provide a powerful decision support tool for medical staff, help optimize the postoperative nursing process, and improve the quality of patient prognosis.

## Data Availability

The raw data supporting the conclusions of this article will be made available by the authors, without undue reservation.
